# Environmental stochasticity controls soil erosion variability

**DOI:** 10.1038/srep22065

**Published:** 2016-03-01

**Authors:** Jongho Kim, Valeriy Y. Ivanov, Simone Fatichi

**Affiliations:** 1 Department of Civil and Environmental Engineering, University of Michigan, Ann Arbor, MI, USA; 2 Department of Civil and Environmental Engineering, Sejong University, Seoul, 143-747, Republic of Korea; 3 Institute of Environmental Engineering, ETH Zurich, Zurich, Switzerland

## Abstract

Understanding soil erosion by water is essential for a range of research areas but the predictive skill of prognostic models has been repeatedly questioned because of scale limitations of empirical data and the high variability of soil loss across space and time scales. Improved understanding of the underlying processes and their interactions are needed to infer scaling properties of soil loss and better inform predictive methods. This study uses data from multiple environments to highlight temporal-scale dependency of soil loss: erosion variability decreases at larger scales but the reduction rate varies with environment. The reduction of variability of the geomorphic response is attributed to a ‘compensation effect’: temporal alternation of events that exhibit either source-limited or transport-limited regimes. The rate of reduction is related to environment stochasticity and a novel index is derived to reflect the level of variability of intra- and inter-event hydrometeorologic conditions. A higher stochasticity index implies a larger reduction of soil loss variability (enhanced predictability at the aggregated temporal scales) with respect to the mean hydrologic forcing, offering a promising indicator for estimating the degree of uncertainty of erosion assessments.

Upland soil erosion by water is one of the essential components for estimating the long-term mass balance of sediment and understanding geomorphological dynamics of basins^1^,^2^. Soil loss from hillslopes, channel, and bank erosion, which in total represent sediment *source*, are balanced by sediment yield at the watershed outlet (*efflux*) and deposition in reservoirs, colluvial foots, or alluvial valleys (*storage*)^2^,^3^,^4^. Measurements or assumptions concerning the efflux and storage allow an indirect estimation of upland soil losses^4^,^5^,^6^. A commonly used alternative is to compute erosion rate directly with models, using information on climate, topography, soil properties, and land use^7^,^8^,^9^,^10^.

Improving assessments and projections of upland soil loss related to land use or climate change has far reaching practical implications. Estimates of upland soil loss have been used as essential input information in a range of analyses, such as identification of the effects of soil erosion on global environmental costs, agricultural productivity, and carbon and nutrient cycling^1^,^7^,^8^,^11^,^12^. What appear to have been the persistent issues connecting erosion science and policy decisions are the reliability of soil loss estimates and their interpretation by both scientists and practitioners^13^,^14^,^15^,^16^. One of the reasons that the estimates obtained with numerical models are repeatedly questioned is because model theories, formulations, and parameters are based on information obtained at relatively small spatial and temporal scales. It is well understood now that heterogeneity, spatial connectivity, and process complexity all increase at larger spatial scales^17^. Furthermore, when considered over longer time periods, the magnitude, frequency of occurrence, and sequence of driving climatological events increase uncertainty of erosion estimates^17^. As such, not only erosion parameters but also soil erosion rates obtained as representative at one set of scales *a priori* cannot be transferred to another scale level^18^,^19^,^20^,^21^.

In erosion research, such a space- and time-scale dependency has long been addressed. The spatial aspect has been explored in numerous experimental studies through scaling from micro to macro domains^19^,^22^,^23^. It is understood now that hydro-geomorphic processes controlling detachment, entrainment, transport and deposition are markedly scale-dependent and thus dominant processes are not unique at different spatial scales. Relatively fewer studies addressed the importance of temporal scale on geomorphic dynamics^19^,^23^, and most of the analyses were based on limited observational periods (<10 years) at a few locations^23^,^24^,^25^. One of the remaining challenges is the observed high temporal non-uniqueness of soil loss given the same rainfall or runoff. The non-uniqueness of soil erosion response can exhibit up to two orders of magnitude difference at various temporal scales^26^,^27^ and thus significantly impacts predictive uncertainty. Profound understanding of involved processes and their interdependencies that contribute to the feature of non-uniqueness has proven to be a challenge. However, it is sorely needed to identify how the environment affects soil erosion and whether there are emerging scaling properties that can be used to inform predictive capabilities across a range of temporal scales.

Using the extensive Universal Soil Loss Equation (USLE^28^), database, (1) we identify temporal scale dependence of soil loss non-uniqueness (also referred to as ‘variability’ hereafter) from unit upland areas. Introducing the notions of erosion *compensation* and soil substrate *stabilization*, (2) we further illustrate that the uncertainty of soil loss is related to stochasticity (the degree of intra- and inter-event variations) of hydrometeorologic conditions. Finally, (3) we derive a novel index that offers the convenience of simplicity and thus can be used in practical, large-scale erosion assessment applications. As a result, we contend that properly informed bounds of uncertainty must accompany soil loss estimate at each scale.

## Hypotheses and Results

### Hypothesis emerging from USLE data

**Figure 1:**
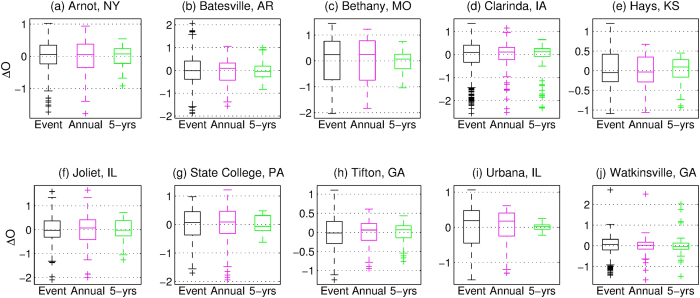
Variability and time scale dependency of the geomorphic response. Boxplots representing residuals from log-linear regressions between runoff and soil loss from the USLE database, expressed as the order of difference (ΔO) at three temporal scales (event, annual, and 5 years) for ten locations. In each boxplot, the central mark is the median, the edges of the box are the 25th and 75th percentiles, and the upper and lower bounds (whiskers) are the maximum and minimum except for outliers (+symbols) that are larger than 1.5 times the interquartile range from the 25th or 75th percentiles.

We use USLE data to derive residuals from log-linear regressions between runoff and soil loss at the storm event, annual, and 5- year scales (Fig. 1 and see *Methods*). The data indicate the high variability/non-uniqueness at the three temporal scales, expressed as the order of difference (ΔO) for all locations. Interesting features that emerge from the USLE data are a reduction of the range of variability for coarser, aggregated temporal scales (Table S1 in the Supplementary Material) and a difference of the reduction degree among the locations (Fig. 1). We propose a hypothesis that stems from these data features: the degree of variability of intra- and inter-event dynamics of hydrometeorologic conditions controls how rapidly non-uniqueness is reduced at larger temporal scale. The hypothesis has direct implications for estimating the uncertainty of soil loss predictability for a given location and across locations.

### Geomorphic ‘compensation’ explains scaling of soil loss variability

**Figure 2:**
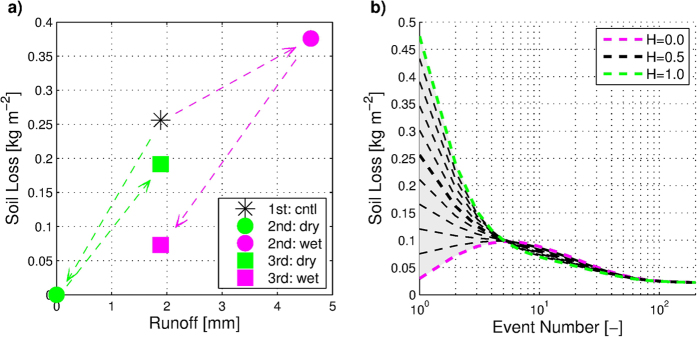
“Compensation” effect and soil substrate “stabilization”. (**a**) “compensation” effect of stochastic variability of hydrometeorologic conditions on soil loss. Soil loss and runoff for three identical consecutive control rainfall events (‘1st’, ‘2nd’, and ‘3rd’, see SM.2.) of which the response to the second event was “perturbed” in terms of antecedent soil moisture condition (Figs S1 and S6a). The green/magenta markers correspond to the simulation results in which antecedent soil moisture is perturbed to a drier/wetter state than that in the 1st, control case (black star). (**b**) Soil substrate stabilization for environment with repeating hydrometeorologic conditions: variations of soil loss are illustrated for 200 successive simulations of event response. The same rainfall forcing and antecedent soil moisture state are assumed for each event. The antecedent particle size distribution (PSD) and the fraction of deposited materials are however iteratively updated as a result of response to preceding rainfall event. *H* stands for the area fraction of highly erodible deposited soil (see Methods).

The proposed hypothesis considers the dependence of variability of geomorphic response on temporal scale and environment. To address this effect, we introduce the notions of soil loss ‘compensation’ and ‘stabilization’. ‘Compensation’ refers to temporal alternation of events that are effective in transporting sediment (i.e., displaying the ‘source-limited’ regime), and those that are mostly limited to breaking down soil matrix, without moving the bulk of perturbed materials (i.e., exhibiting the ‘transport-limited’ regime). Due to the stochastic nature of variations of hydrometeorologic conditions, the chance that the occurrence of one type of event is ‘compensated’ by that of the other grows with temporal scale. This, for example, refers to the chance of occurrence of lower or higher rainfall, drier or wetter antecedent moisture conditions, which all exhibit different chances of runoff production and overland flow occurrence. Using two scenarios (see *Methods*), Fig. 2a illustrates how moderate perturbations of antecedent soil moisture, replicating the differences in interstorm drying, result in initially large differences in soil loss, which are later ‘compensated’ by a subsequent event (see also Fig. S1). A similar compensatory behavior can be achieved when different rainfall forcings are applied to identical antecedent moisture conditions (Fig. S2).

The physical reasons for such dynamics become clear if one examines the evolution of the soil substrate composition. For instance, the drier antecedent condition in Fig. 2a leads to complete infiltration of rainfall and zero runoff and soil loss, but it also results in a high fraction of materials splashed by rainfall on the soil bed (Fig. S3), which are readily mobilized by the subsequent runoff event. In contrast, the wetter initial state causes detachment and entrainment of relatively lighter particles with overland flow that leaves behind coarser soil material. The particle distribution determines surface erodibility conditions, and thus the geomorphic response to the next runoff event. It follows that the relation between climatic variability, such as that of rainfall or evaporative strength during interstorm periods (external forcing), and erosion response is mediated by the micro-scale soil surface conditions.

### Soil ‘stabilization’ and disruption in environments with low stochasticity

When the variability in the micro-scale erodibility is considered in the context of stochasticity of hydrometeorologic forcing, it is logical that longer temporal scales should enhance the likelihood of the ‘compensation’ effect. Therefore, an increase of the covariance of mean runoff and sediment loss and thus a reduction of the non-uniqueness are expected. Such a trend can be observed for most of the USLE sites, however, for some locations the degree of the variability reduction at coarser time scales is not that prominent (e.g., Arnot and Joliet in Fig. 1). This is best understood if one analyzes the dependence of the ‘compensation’ effect on temporal scale. To be a valid concept, the considered scale should be such that the probabilities of occurrence of ‘transport-limited’ and ‘source-limited’ events multiplied by the respective values of expected sediment yield are comparable. The ‘source-limited’ type is due to infrequent, extreme hydrologic conditions, which are progressively likelier to occur when the temporal window of integration is increased. However, in hydro-environments with low stochastic variability, a threshold scale at which ‘compensation’ can be considered to be valid appears to exceed the conventional scales of analysis (few years). We therefore hypothesize that a contributing reason to the persistence of variability is the weak stochasticity of the hydro-environmental forcing or, in other words, the lack of extreme ‘source-limited’ events in the observation period, which leads to a ‘quasi-stabilization’ of soil substrate, as explained in the following.

To illustrate the contributing effect of ‘stabilization’, recursive simulations (see *Methods*) are carried out that use identical rainfall with the same initial hydrologic condition, except for antecedent soil substrate state that is continuously updated (Fig. 2b and Fig. S4). The results reveal that periodic forcing (i.e., zero stochasticity) leads to stabilization of the evolution of deposited soil layer in terms of its spatial fraction and particle size distribution (Fig. S4). Stabilization is also maintained when a perturbation in the rainfall forcing or soil moisture condition is introduced at some later stage (Fig. S5), providing evidence that the same steady-state can be achieved under identical or very similar cyclic boundary condition. Stable composition of soil bed and constant surface erodibility is a theoretical limiting situation that cannot be found in the real cases under consideration. Nevertheless, one may hypothesize that in hydro-environments with weak stochasticity, ‘quasi-stable’ surface conditions corresponding to a steady-state, (i.e., narrowly constrained ranges of PSD and erodibility conditions) are possible for periods of time. They are however interrupted by infrequent, atypical geomorphic events that force the system to deviate from its quasi-steady-state and thus introduce perturbations to the mean response. This causes a subsequent period of transient geomorphic response (e.g., Fig. S5), contributing to the explanation of the weak dependence of soil loss variability with increasing temporal scale.

### Environment stochasticity and variability of geomorphic response

If the proposed concept of the ‘compensation’ effect is correct, higher stochasticity (larger relative entropy, see *Methods*) of hydrometeorologic conditions should lead to a higher chance for compensation and thus a larger reduction in geomorphic response variability at coarser temporal scales. Very small stochasticity of hydrometeorologic conditions (small relative entropy) can be viewed as the driving cause for system quasi-stabilization, implying that variability of geomorphic response at different temporal scales is comparable and generally smaller than that in environments with high stochasticity. Between the two limiting cases, one can expect a range of dynamics that should exhibit a correlation between the reduction of geomorphic response variability across aggregation scales and the degree of stochasticity of hydro-environment.

**Figure 3:**
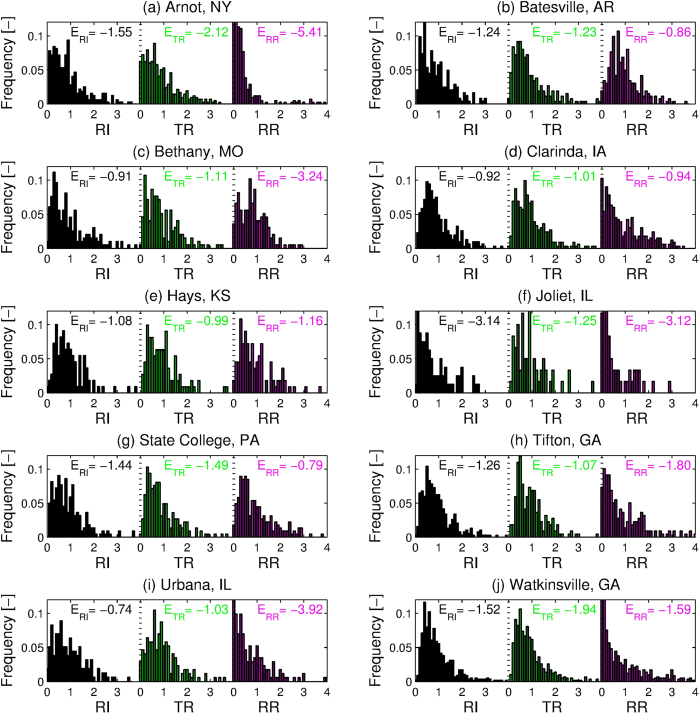
Stochasticity of hydrometeorologic conditions. Frequency distributions of 30-minute rainfall intensity (*RI*), total rainfall volume (*TR*), and runoff ratio (*RR*) normalized by the respective averages over all runoff events recorded in the USLE database. Estimates of the corresponding magnitudes of relative entropy ‘*E*_*RI*_’, ‘*E*_*TR*_’, and ‘*E*_*RR*_’ are provided in the subplots.

**Figure 4:**
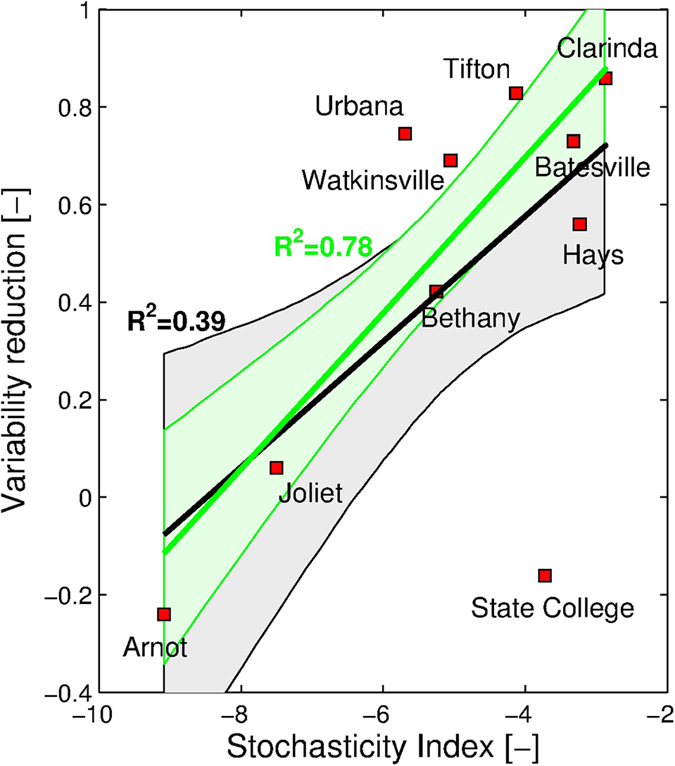
A relationship between the stochasticity index (the sum of relative entropy values) and reduction in geomorphic variability. The reduction of soil loss variability is taken as the difference of boxplot ranges (upper – lower bounds) in Fig. 1 computed for the event and annual scales. The coefficient of determination is reported for linear regression that includes all data (black) and excludes ‘State College’ data point (green) - the location with the shortest period of observations. The grey and light green shaded areas represent the 95% confidence intervals (from 5,000 samples of bootstrapped data) of the black and green regression lines, respectively.

Stochasticity of the hydro-environment can be characterized by exploring the degree of variations in the two primary forcings affecting the geomorphic response - rainfall and runoff. To indicate the overall characteristics of hydrometeorologic conditions at a given location, we propose a stochasticity index (see *Methods*), which is defined as the sum of three relative entropy indices for the variables of observed 30-minute rainfall intensity (*RI*), total rainfall volume (*TR*), and runoff ratio (*RR*) that represent rainfall intensity, rainfall amount, and a scaled measure of the hydrological response, respectively. The relative entropy quantifies the extent of stochastic variability of each variable: it is zero in environments with high stochastic variability and is maximized in absolute value for environments with homogeneous conditions. For example, as can be concluded from Fig. 3, the probability distributions of *RI*, *TR*, and *RR* for the location Clarinda, IA, exhibit higher stochasticity and thus their entropy magnitudes are relatively closer to zero when compared to the other locations, such as Arnot, NY. The stochasticity index computed for the USLE locations is positively related to the reduction of soil loss variability between event and annual scales (Fig. 4). This provides a distinct quantitative support for the hypothesis of this study based on a large set of empirical data.

## Discussion

The observed phenomenon of the reduction of soil loss variability at aggregated temporal scales is attributed to the ‘compensation effect’, which is due to the stochastic variations of hydrometeorologic forcing, causing alternation of conditions that suppress or enhance erosion for subsequent events. In other words, climate and soil substrate conditions result in stochastic sequences of ‘transport-’ or ‘source-limited’ geomorphic regimes. It is consistent to expect that longer temporal scales should increase the chance of the hypothesized ‘compensation effect’ because geomorphic system can experience a larger number of possible contrasting regimes. However, it is also clear that some sites exhibit a less pronounced decay of soil loss variability at larger temporal scales. This is attributed to a system predisposition to achieve a preferred geomorphic regime stabilizing its particle size distribution, which is intermittently disrupted, leading to a less efficient compensatory regulation.

What are the practical implications? Due to the overall high variability of soil loss with respect to runoff at typical time scales of empirical observation, erosion predictive skill is likely to remain handicapped even in when accurate runoff data are available. An optimistic view on the problem is however that the skill appears to be asymptotically consistent for larger temporal scales, albeit the skill convergence will depend on site location, as illustrated here. In general, policy makers or managers have already relied on long-term estimate of soil loss. For example, the U.S. Department of Agriculture developed the National Resources Inventory (NRI^29^) that uses the Universal Soil Loss Equation – an empirical equation developed based on *long-term* erosivity (rainfall) and erodibility (soil) factors. The effect of variability reduction identified here therefore ensured that past decisions of long-term planners have been already cushioned, to some extent, from the higher uncertainty that accompany estimates from data collected over shorter periods. The missing element however remains an explicit inclusion of uncertainty into the assessment equations. One possible approach could be the development of a probabilistic framework that may contemplate ideas from the hydrologic frequency analysis of extremes or landslide occurrence, such as events of specific recurrence intervals and the probability of occurrence of soil loss corresponding to a given percentile range. Such probabilistic approaches would contribute to more appropriate planning tools for long-term assessments.

We introduce a stochasticity index, which expresses the degree of intra- and inter-event variations of environmental conditions. The reduction of geomorphic response variability from the event to the annual scales is linearly (positively) related to the index, corroborating the hypothesis that the high stochasticity of hydrometeorologic forcing increases the chance of the ‘compensation effect’ and reduce uncertainty in the long-term. Conversely, weak stochasticity of the forcing increases the temporal scales at which the ‘compensation effect’ can be considered valid. A practical implication is that the stochasticity index could be a promising parsimonious indicator in estimating the degree of assessment uncertainty, when only a single average soil loss estimate is used in a management decision. However, the developed relationship (Fig. 4) is derived through an analysis of ten experimental sites that are only located in agricultural fields in the U.S. To enhance robustness of index-based applications, we recommend the following course of action be undertaken. Data bank integrating all existing observations on soil loss and vital auxiliary characteristics should be developed for upland and headwater areas. The entire USLE data set as well as other long-term soil loss monitoring data from other environmental settings should be released/shared within the community. Long-term data on soil loss from unit upland and headwater areas need to continue to be collected.

## Methods

### Universal Soil Loss Equation database

The USLE database contains storm characteristics (intensities, amount, duration, etc.), runoff, soil loss, and site-specific description. The data consist of over 11,000 plot-years of observations at 47 locations in the U.S. The current availability is however limited to 3,195 plot-years (310 individual plots) at 14 field sites (http://topsoil.nserl.purdue.edu/usle). Many locations contain observations made for replicated plots, the number of which varies over time. For such plots, experimental conditions in terms of hydrometeorologic forcing, plot slope, soil type, and landuse were identical. Data from these replicated plots were included in the analysis because the data represent possible variability of soil loss within a plot that is subject to the same climate forcing and land conditions. After elimination of events for which plot replications were not consistently used, the analysis data set used here includes 884 erosion events for 10 locations (1218 plot-years, 102 individual plots), with the number of replicated plots among given locations varying between 4 and 16. Calendar years are used to compute averages at the annual aggregation scale. To estimate values at the 5-year aggregation scale, moving average over five consecutive calendar years is carried out resulting in correlated 5-year averages (i.e., averaging window is moved forward by one year each time 5-year value is computed). The order of magnitude difference (ΔO) is computed as the difference of the common logarithm of data values and values obtained from log-linear regression between runoff and soil loss.

### Coupled numerical model

The tRIBS-VEGGIE-FEaST (Triangulated irregular network - based Real time Integrated Basin Simulator- VEGetation Generator for Interactive Evolution -Flow Erosion and Sediment Transport) is used. The model partitions energy budget at surface^30^,^31^ and represents subsurface saturated-unsaturated zone dynamics using the 1-D Richards and Boussinesq equations under the Dupuit-Forchheimer assumptions^32^. The overland component solves a system of governing equations of 2-D Saint-Venant and Hairsine-Rose equations for surface flow and sediment movement using a holistic, physically-consistent approach^33^,^34^,^35^. One of the key features of the model is dynamic simulation of area fraction (*H*) of highly erodible deposited soil and that (1-*H*) of original, “intact” soil, in which contact forces hold particles together. While the soil surface is allowed to change during the simulation due to the local processes of erosion and deposition, at the event-scale, the feedback of change in elevation and slope to hydrologic processes is weak. Therefore, one-way coupling between hydrology and erosion processes is used in the study.

Numerical representation of the domain (size, slope, and mesh properties), the boundary and initial conditions for surface and subsurface domains, soil properties (type, composition, settling velocities, and hydraulic characteristics), and details on numerical experiments for Figs 2 and S1, S2, S4, and S5 are provided in Supplementary Material.

### Stochasticity index

Frequency distributions of observed 30-minute rainfall intensity (*RI*), total rainfall volume (*TR*), and runoff ratio (*RR*), as reported in the USLE database, normalized by the respective average values were computed (Fig. 3). The three normalized metrics representing the intensity and amount of rainfall, and hydrological response indicate the overall characteristics of hydrometeorologic conditions at a given location. An entropy index for each site is defined using the concept of ‘relative entropy’^36^, the latter estimated for the three metrics (Fig. 3). Specifically, the relative entropy is computed as 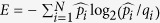, where 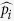 is the sample probability density of a given metric associated with the interval *i*, and *q*_*i*_ is the probability density for the uniform distribution; 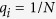 for all intervals, where *N* is the number of intervals obtained as the metric maximum divided by 0.1. The relative entropy is zero if 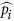 follows the uniform distribution (i.e., high stochastic variability). Conversely, if the density approaches the Dirac delta function, the absolute relative entropy is maximized (i.e., environment with uniform conditions). The stochasticity index is the sum of the three relative entropy estimates and thus represents the degree of stochasticity of hydrometeorologic conditions at a given location: smaller index values indicate more uniform conditions, while larger values imply a larger degree of fluctuations over the long-term.

## Additional Information

**How to cite this article**: Kim, J. *et al*. Environmental stochasticity controls soil erosion variability. *Sci. Rep*. **6**, 22065; doi: 10.1038/srep22065 (2016).

## Supplementary Material

Supplementary Information
