# Supplementary material for: Environmental stochasticity controls soil erosion variability
Source: Sci Rep. 2016 Mar 1;6:22065. doi: 10.1038/srep22065 (PMC4771998; doi:10.1038/srep22065)
Supplement: Supplementary Information [file srep22065-s1.pdf]

Supplementary Material to

"Environmental stochasticity controls soil erosion variability"

Jongho Kim[1,2], Valeriy Y. Ivanov[1], and Simone Fatichi[3]

[1]Department of Civil and Environmental Engineering, University of Michigan, Ann Arbor, MI

[2]Department of Civil and Environmental Engineering, Sejong University, Seoul, Republic of Korea

[3]Institute of Environmental Engineering, ETH Zurich, Zurich, Switzerland

Corresponding author: Valeriy Y. Ivanov, Department of Civil and Environmental Engineering, University of Michigan, Ann Arbor, MI 48109, tel: 734-763-5068, email: ivanov@umich.edu.

This file includes:

Methods: SM.1 to SM.2

Figs. S1 to S6

Table S1 to S2

Reference list

SM.1. Numerical modeling

The tRIBS-VEGGIE-FEaST model (see *Methods*) was applied to a hillslope plot located near Clarinda, IA, USA, for which a large number of erosion events (312 in total) replicated over 9 plots has been observed over a long-term period (32 years). Experimental areas are 'unit plots' of rectangular shape with width and length equal to 1.8 m and 22.1 m, respectively, and slope equal to 9 %. To be represented in the numerical model, the plot surface was spatially discretized with a triangular mesh, composed of equilateral triangles of 0.0167 m^2 area. The plot subsurface was resolved with irregularly spaced mesh (18 nodes) at a spatial step that varied from 0.02 m for the top soil layer to 0.1 m for the bottom layer (1 m depth). Bare soil condition was specified. Open boundary (at downslope) and no-flux boundary (all other sides of the domain) conditions were specified for surface flow. Flux (surface) and zero flux (bedrock interface) boundary conditions were assumed for subsurface flow. Hydrostatic distribution (for soil moisture) and dry condition (for surface flow) were assumed as initial conditions. Only a minimal model calibration was carried out to confirm response magnitude. It resulted in the simulated runoff volume that was similar to average runoff of the nine replicate plots.

A rainfall event representative of the location of Clarinda, i.e., the one that resulted in the mean runoff and soil loss, was selected among 312 events. Using observed storm statistics reported in the USLE database, continuous, representative 15-minute interval rainfall time series for the event (the control time series in Fig. S6b) was obtained with a stochastic disaggregation technique, specifically, the multiplicative, microcanonical method [*Molnar and Burlando*, 2005; *Paschalis et al.*, 2014]. The generated time series completely satisfied the reported duration, total depth, as well as 5, 15, 30, and 60 minute interval peak intensities.

The plot soil type is Marshall silty clay loam [*USDA*, 1965], which consists of clay, silt, and sand with fractions of 32.4, 64.1, and 3.5 [%], respectively. For numerical purposes, the soil was

characterized with six particle sizes of 0.002, 0.004, 0.01, 0.03, 0.05, and 0.1 [mm], each of which has fractions of 16.2, 16.2, 21.36, 21.36, 21.36, 3.52 [%], respectively. The smallest particle type is called P1, the second smallest and progressively larger particle types are denoted as P2 through P6. Their corresponding settling velocities, $\nu_{1,\cdots,6}$, are 0.00000277, 0.0000111, 0.0000692, 0.000617, 0.0017 and 0.0062 [m s^{-1}] are calculated using a formula of *Cheng* [1997]. The total fraction of the deposited mass was also varied from completely intact (H = 0), to entirely loose soil (H = 1), with an increment of 0.1. The values of soil hydraulic parameters were estimated using the ROSETTA model (http://www.ars.usda.gov/News/docs.htm?docid=8953#references). The values of flow and erosion parameters were inferred from previous studies. The parameter values are provided in Table S2.

SM.2. Numerical experiments for Figs. 2, S1, S2, S4, and S5

In Fig. 2**a** (main text, also Fig. S1 here), soil loss and runoff for contrasting antecedent soil moisture conditions are illustrated. Three identical consequent rainfall events were used, of which only the second event was “perturbed” to a drier/wetter state than that in the control case. Fig. S6**a** illustrates how drier and wetter conditions were generated. Similarly, Fig. S2 corresponds to simulation results for which rainfall forcing was specified as two times smaller/larger than that in the control case (Fig. S6**b**). In Fig. 2**b** (also Fig. S4), 200 ensemble simulations for three PSDs of the deposited layer were carried out by using the same antecedent soil moisture state and rainfall forcing. (marked as, ‘midSL’, ‘maxSL’; the ‘minSL’ and ‘maxSL’ compositions correspond to the minimum (‘minSL’ and maximum amount of soil loss for each scenario of H fraction. The ‘midSL’ has the same PSD as the original, intact soil layer) Antecedent soil substrate conditions were iteratively updated 200 times as response to consecutive rainfall events. Four additional 200 ensemble simulations for the ‘midSL’ PSD (Fig. S5) were performed by keeping all forcings and

conditions unchanged, except for perturbing the second event with a rainfall volume that was smaller or larger (and soil state - drier or wetter) than in the control case (middle plots in Fig. S4).

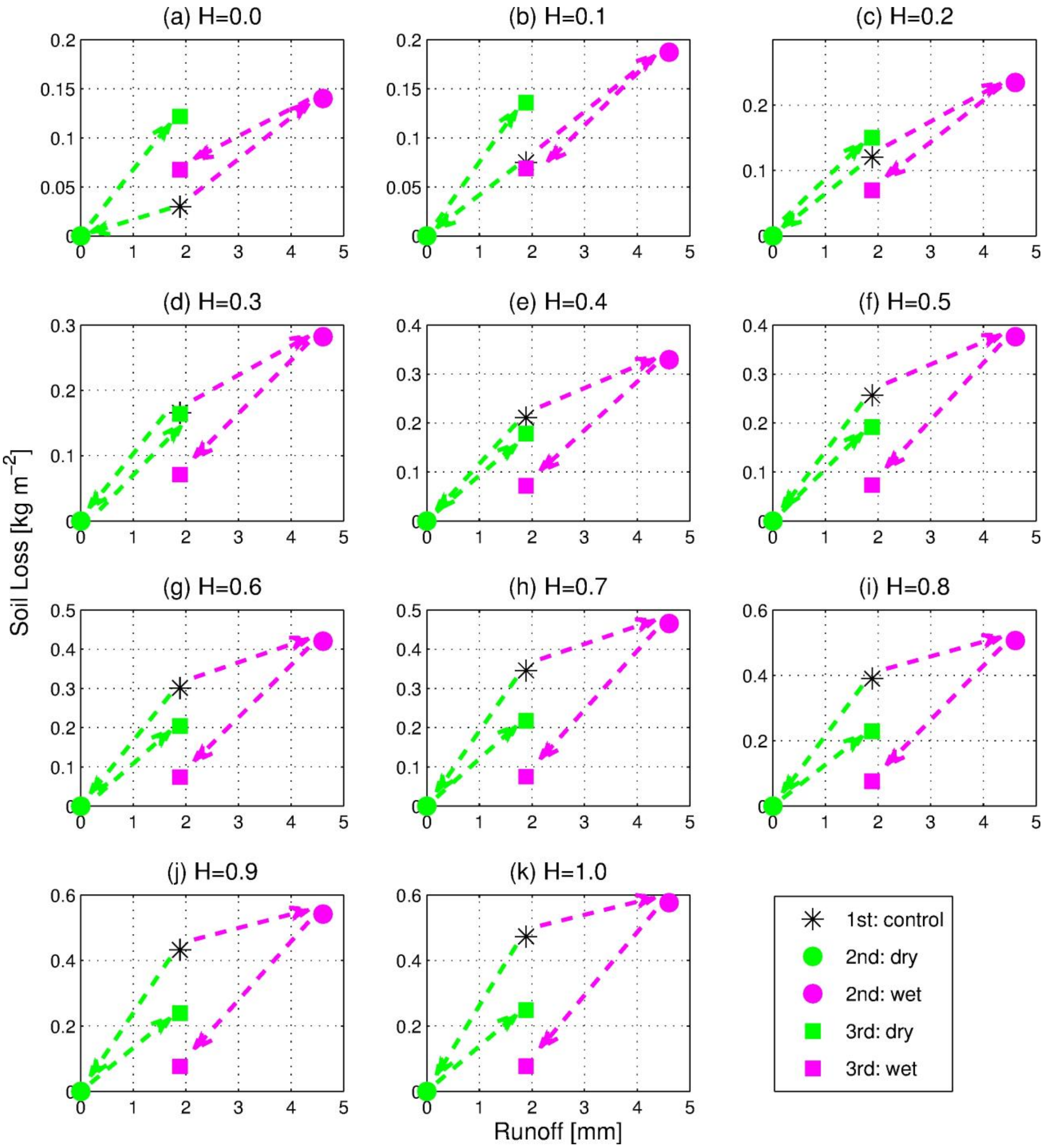

Figure S1 | "Compensation effect" of stochastic variability of hydrometeorologic conditions on soil loss. Soil loss and runoff for 3 identical consequent control rainfall events ('1st', '2nd', and '3rd') of which the response to the second event was "perturbed" in terms of antecedent soil moisture condition. The green/magenta markers correspond to the simulation results in which antecedent soil moisture is perturbed to a drier/wetter state (Fig. S6a) than that in the 1st, control case (black star). The results were obtained using the "midSL" particle size distribution with initial *H* values indicated in the subplot titles.

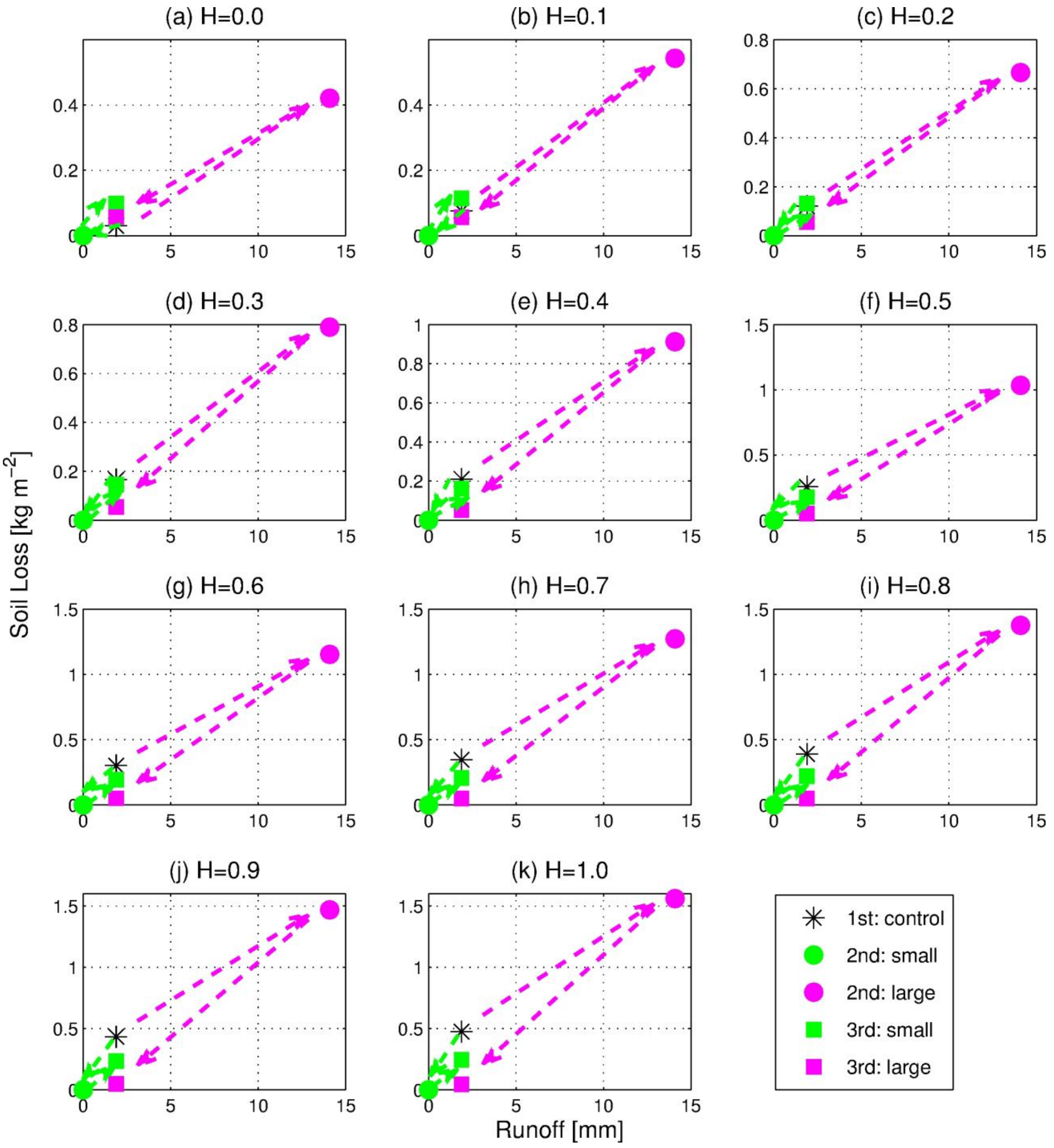

Figure S2 | "Compensation effect" of stochastic variability of hydrometeorologic conditions on soil loss. Soil loss and runoff for 3 identical consequent control rainfall events ('1st', '2nd', and '3rd') of which the response to the second event was "perturbed" in terms of rainfall forcing. The green/magenta markers correspond to the simulation results in which rainfall is changed to a smaller/larger magnitude (Fig. S6b) than that in the 1st, control case (black star). The results were obtained using the "midSL" particle size distribution with initial *H* values indicated in the subplot titles.

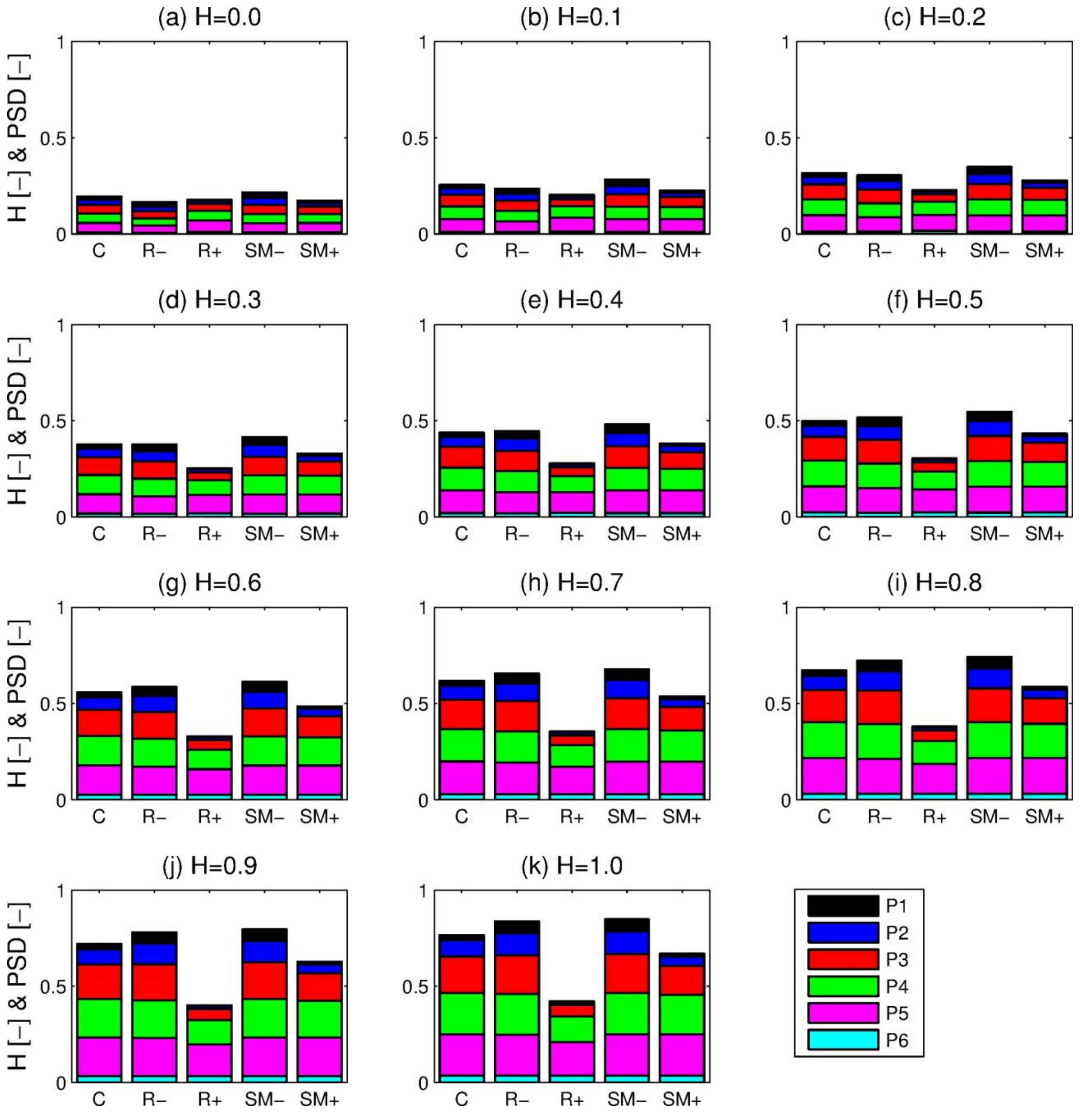

Figure S3 | Post-event soil substrate composition. Average spatial fraction (*H*) and composition (i.e., PSD) of post-event deposited layer for ten values of antecedent *H* (indicated in sub-plot titles). The symbols refer to control ('C'), smaller ('R-'), and larger ('R+') rain, drier ('SM-'), and wetter ('SM+') soil moisture conditions. The results correspond to the soil substrate conditions after the second event illustrated in **a-k** in Fig. S1 and S2. The 'midSL' initial PSD was used in all simulations.

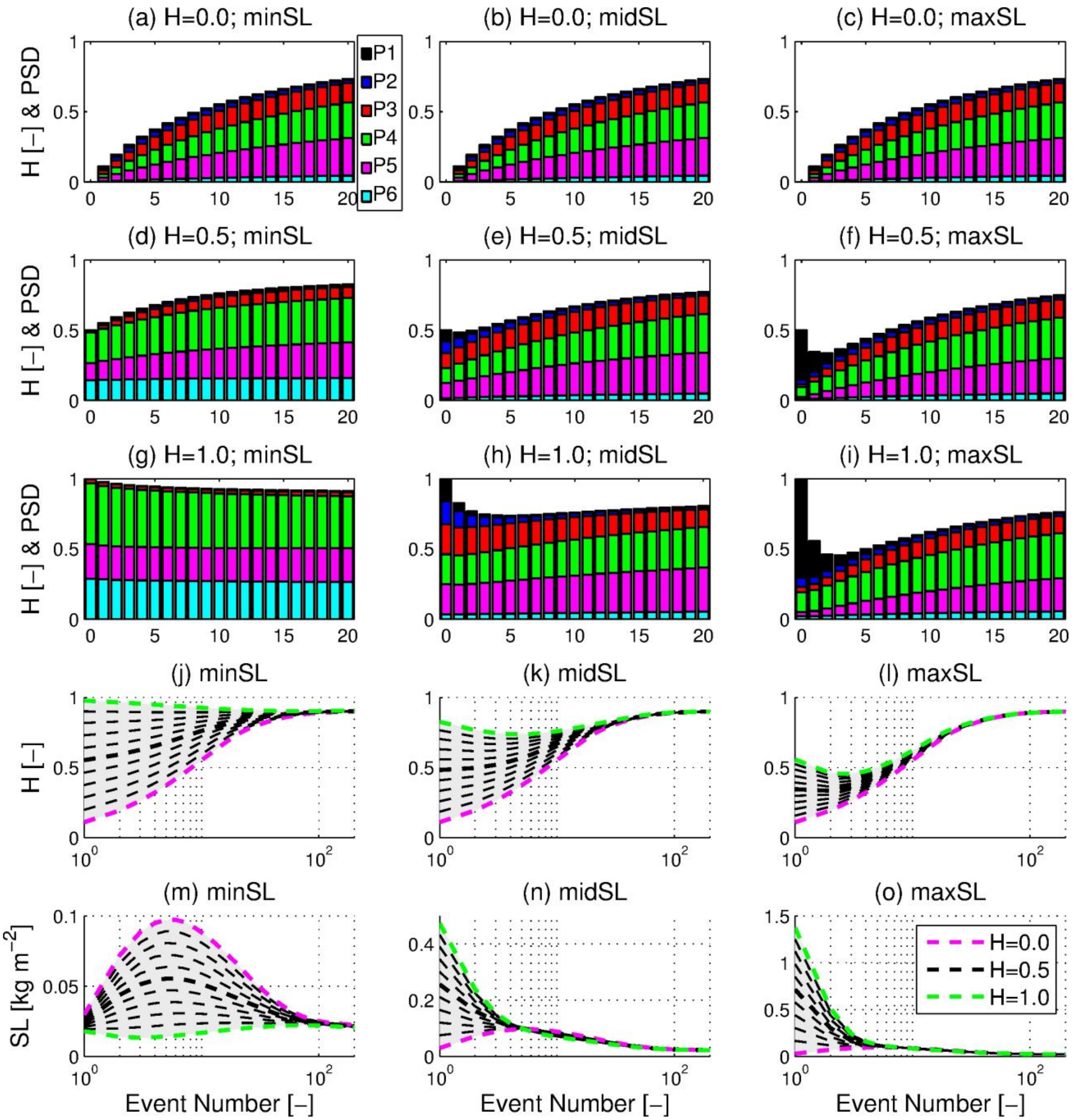

Figure S4 | Soil substrate stabilization for environment with cyclic hydrometeorologic conditions. Ensemble variations of the fractional magnitude (*H*) and composition (PSD) of the deposited layer and soil loss (*SL*) for three initial PSDs: minSL (left plots), midSL (middle plots), and maxSL (right). 200 ensemble simulations were carried out by using the same antecedent soil moisture state and rainfall forcing (*H* varying from 0 to 1.0, at a resolution of 0.1, is used in the first simulation). Antecedent soil substrate conditions were iteratively updated by response to preceding rainfall event, **a-o** (20 ensemble simulation results out of 200 are shown for clarity in **a-i**).

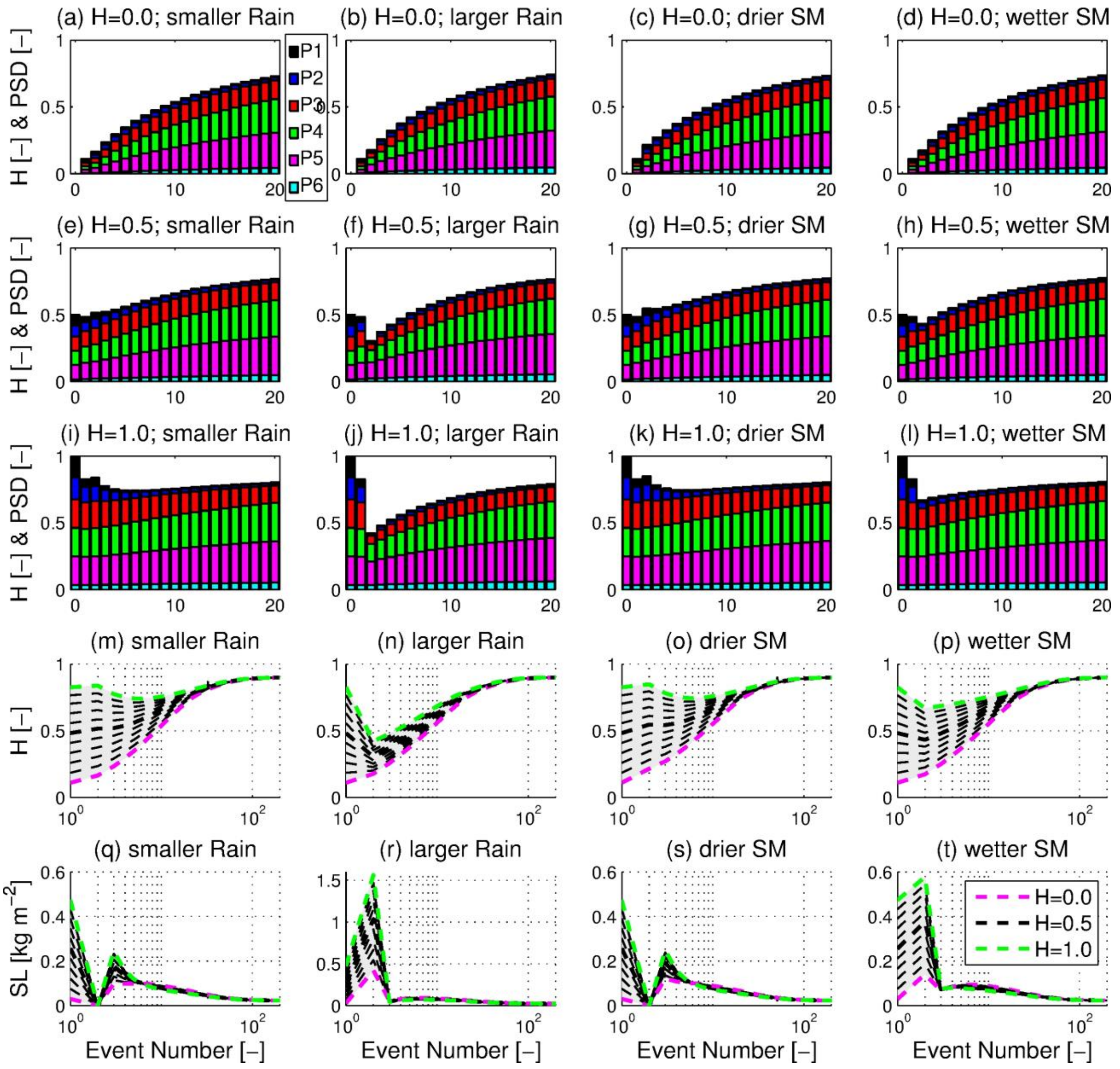

Figure S5 | Convergence to the same steady-state under cyclic boundary conditions. Ensemble variations of amounts (*H*) and compositions (PSD) of deposited layer and soil loss (*SL*) for the 'midSL'. Four additional 200 ensemble simulations were carried out by using the same antecedent soil moisture state and rainfall forcing (*H* varying from 0 to 1.0, at a resolution of 0.1, is used in the first simulation), with the exception of an introduced perturbation of rainfall volume to a smaller (the column of plots on the left) or larger (the 2nd column) magnitude; or soil moisture to a drier (the 3rd column) or wetter (4th column) initial state than that in the control case. 20 ensemble simulation results out of 200 are shown for clarity in **a-l**.

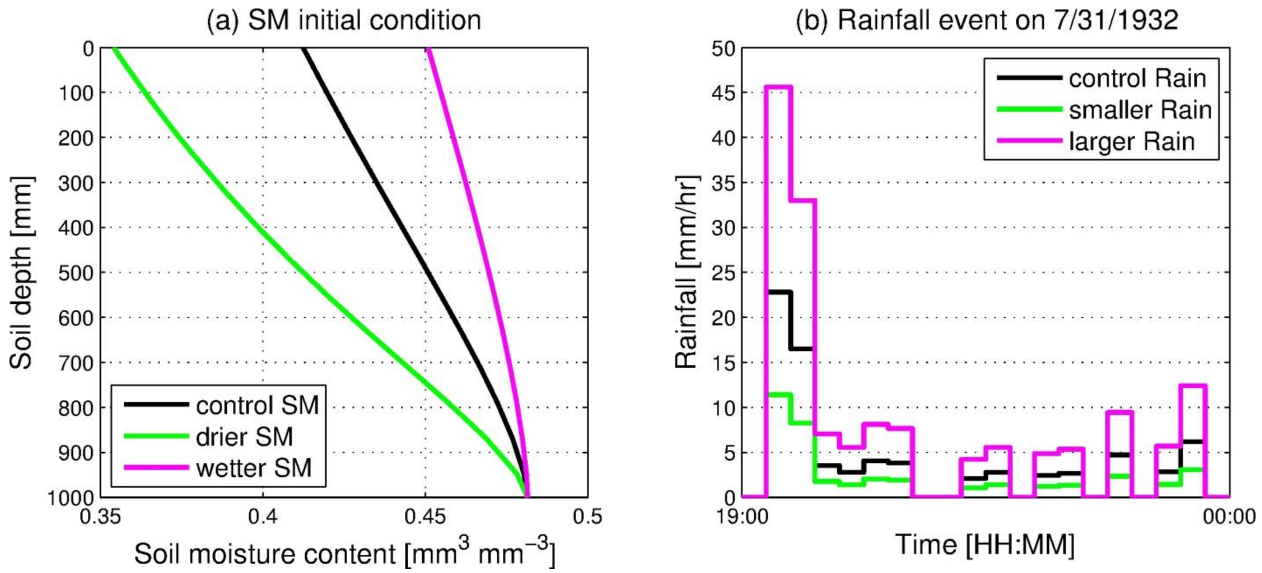

Figure S6 | Synthetic modifications of initial soil moisture condition and rainfall forcing. a, The black line is the control soil moisture distribution corresponding to the hydrostatic condition in soil with the saturation condition at 1 m depth and α (of the van Genuchten's soil hydraulic parameterization) equal to 0.0008. The green/magenta lines correspond to the drier/wetter soil moisture conditions generated by using values of α that are 2 times larger/smaller than that in the control case. **b,** the rainfall time series disaggregated at 15-minute intervals. As compared to the rain control case, the perturbed rainfall series have two times smaller/larger rate. The perturbed condition (soil moisture or rainfall) was specified only for the second event in Fig. S1, S2 and S5.

Table S1. Homogeneity test p-values for ten USLE locations: (a) Arnot, NY; (b) Batesville, AR; (c) Bethany, MO; (d) Clarinda, IA; (e) Hays, KS; (f) Joliet, IL; (g) State College, PA; (h) Tifton, GA; (i) Urbana, IL; (j) Watkinsville, GA. The null hypothesis is that the variances of soil loss of the three groups for different temporal scales (i.e., event, annual, and 5-year) are equal.

Test	(a)	(b)	(c)	(d)	(e)	(f)	(g)	(h)	(i)	(j)
**Brown-Forsythe**[1]	5.05 E-02	3.36 E-04	1.06 E-06	3.97 E-10	5.07 E-02	4.38 E-02	1.78 E-03	7.39 E-10	1.59 E-06	2.58 E-04
**Levene**[2]	4.16 E-02	3.76 E-04	4.81 E-09	1.01 E-09	5.21 E-02	3.43 E-02	1.07 E-03	8.04 E-09	3.39 E-10	1.36 E-04

[1] Brown, M.B. and Forsythe, A.B. (1974) Robust tests for the equality of variances. Journal of the American Statistical Association 69(346), 364-367.

[2] Levene, H. (1960) Robust tests for equality of variances. In Ingram Olkin, Harold Hotelling, et alia. Contributions to Probability and Statistics: Essays in Honor of Harold Hotelling. Stanford University Press., 278-292.

Table S2. Parameters used to represent hydrologic, hydraulic, and sediment erosion-transport dynamics for Clarinda, IA.

Description	Value	Unit	Source	Usage
Manning coefficient	0.03	$s\ m^{-1/3}$	*Kim and Ivanov* [2014]	Flow
Detachability of original soil	20	$kg\ m^{-3}$	*Kim and Ivanov* [2014]	Erosion
Detachability of deposited soil	2000	$kg\ m^{-3}$	*Kim and Ivanov* [2014]	Erosion
Effective fraction of excess stream power	0.01	-	*Kim and Ivanov* [2014]	Erosion
Critical stream power	0.0439	$W\ m^{-2}$	*Heng et al.* [2011]	Erosion
Specific energy of entrainment	750	$m^2\ s^{-2}$	*Heng et al.* [2011]	Erosion
Deposited mass needed to sheild original soil	2.7	$kg\ m^{-2}$	*Kim and Ivanov* [2014]	Erosion
Saturated hydraulic conductivity	4.279	$mm\ hr^{-1}$	ROSETTA	Soil-hydraulic
Volumetric soil moisture at saturation	0.4815	$m^3\ m^{-3}$	ROSETTA	Soil-hydraulic
Volumetric residual soil moisture	0.0913	$m^3\ m^{-3}$	ROSETTA	Soil-hydraulic
Pore-size distribution index	1.5093	-	ROSETTA	Soil-hydraulic
Air entry bubbling pressure	-0.00085	mm	ROSETTA	Soil-hydraulic

References

Cheng, N. (1997), Simplified settling velocity formula for sediment particle, *Journal of Hydraulic Engineering-ASCE*, *123*(2), 149-152.

Heng, B. C. P., G. C. Sander, A. Armstrong, J. N. Quinton, J. H. Chandler, and C. F. Scott (2011), Modeling the dynamics of soil erosion and size-selective sediment transport over nonuniform topography in flume-scale experiments, *Water Resources Research*, *47*.

Kim, J., and V. Y. Ivanov (2014), On the nonuniqueness of sediment yield at the catchment scale: The effects of soil antecedent conditions and surface shield, *Water Resources Research*, *50*(2), 1025-1045.

Molnar, P., and P. Burlando (2005), Preservation of rainfall properties in stochastic disaggregation by a simple random cascade model, *Atmospheric Research*, *77*, 137-151.

Paschalis, A., P. Molnar, S. Fatichi, and P. Burlando (2014), On temporal stochastic modeling of precipitation, nesting models across scales, *Advances in Water Resources*, *63*, 152-166.

USDA (1965), Predicting rainfall-erosion losses from cropland east of the rocky mountains: Guide for selection of practices for soil and water conservation, *Agriculture Handbook*, *282*.
